# Associations of 2D speckle tracking echocardiography-based right heart deformation parameters and invasively assessed hemodynamic measurements in patients with pulmonary hypertension

**DOI:** 10.1186/s12947-020-00197-z

**Published:** 2020-05-14

**Authors:** Lena Theres, Anne Hübscher, Karl Stangl, Henryk Dreger, Fabian Knebel, Anna Brand, Bernd Hewing

**Affiliations:** 1 Medizinische Klinik m.S. Kardiologie und Angiologie, Charité-Universitätsmedizin, Campus Mitte, Charitéplatz 1, 10117 Berlin, Germany; 2 DZHK (German Center for Cardiovascular Research), partner site, Berlin, Germany; 3 Berlin Institute of Health (BIH), Berlin, Germany; 4 Zentrum für Kardiologie, Kardiologische Gemeinschaftspraxis, Muenster, Germany; 5 Department of Cardiology III - Adult Congenital and Valvular Heart Disease, University Hospital Muenster, Muenster, Germany

**Keywords:** Right atrial strain, Right ventricular strain, Pulmonary hypertension, 2D speckle tracking echocardiography, Post-capillary pulmonary hypertension, Hemodynamic

## Abstract

**Background:**

We aimed to evaluate associations of right atrial (RA) and right ventricular (RV) strain parameters assessed by 2D speckle tracking echocardiography (2D STE) with invasively measured hemodynamic parameters in patients with and without pulmonary hypertension (PH).

**Methods:**

In this study, we analyzed 78 all-comer patients undergoing invasive hemodynamic assessment by left and right heart catheterization. Standard transthoracic echocardiographic assessment was performed under the same hemodynamic conditions. RA and RV longitudinal strain parameters were analyzed using 2D STE. PH was defined as invasively obtained mean pulmonary arterial pressure (mPAP) ≥25 mmHg at rest and was further divided into pre-capillary PH (pulmonary capillary wedge pressure [PCWP] ≤ 15 mmHg), post-capillary PH (PCWP > 15 mmHg) and combined PH (PCWP > 15 mmHg and difference between diastolic PAP and PCWP of ≥7 mmHg). Correlation analyses between variables were calculated with Pearson’s or Spearman’s correlation coefficient as applicable.

**Results:**

Out of 78 patients, 45 presented with PH. Within the PH group, 39 had post-capillary, five had combined pre- and post-capillary PH, and one had pre-capillary PH. Patients with PH had a significantly increased RA area (PH 22.0 ± 9.2 cm^2^, non-PH 17.3 ± 10.7 cm^2^; *p* = 0.003) and end-systolic RV area (PH 14.7 ± 6.1, non-PH 11.9 ± 4.8 cm^2^; *p* = 0.022). RV mid strain was significantly reduced in PH (PH -17.4 ± 7.8, non-PH: − 21.6 ± 5.5; *p* = 0.019). Average peak systolic RA strain (RAS) and average peak systolic RV strain (RVS) showed a significant association with mPAP (r = − 0.470, *p* = 0.001 and r = 0.490, p = 0.001, respectively) and with PCWP (r = − 0.296, *p* = 0.048 and r = 0.365, *p* = 0.015, respectively) in patients with PH. Furthermore, RV apical, mid and basal strain as well as RV free wall strain showed moderate associations with mPAP. In patients without PH, there were no associations detectable between RA or RV strain parameters and mPAP and PCWP.

**Conclusion:**

In an all-comer cohort, RA and RV strain parameters showed significant associations with invasively assessed mPAP and PCWP in patients with predominantly post-capillary PH. These associations may be useful in clinical practice to assess the impact of post-capillary PH on myocardial right heart function.

## Introduction

While diagnosis of PH is based on invasive hemodynamic measurements of pulmonary artery pressure (PAP) by right heart catheterization, transthoracic echocardiography (TTE) remains the widely available screening and monitoring tool for PH. According to current PH guidelines [1], estimation of systolic PAP is recommended in patients with tricuspid regurgitation by Doppler Imaging. Recommendations, however, on functional assessment of RA and RV impairment due to PH remain scarce. Recent studies have shown that RA and RV dysfunction are important prognostic factors and indicators for adverse outcome in patients with pre-capillary PH [2–5]. 2D STE reportedly constitutes a feasible additional tool for the assessment of RA and RV function [6, 7] and normative reference data for RA and RV 2D STE have been published for healthy populations [8–10]. Furthermore, the prognostic value of RA and RV strain parameters has been evaluated in patients with pre-capillary PH [11, 12]. However, studies assessing the association of RA and RV mechanics with invasively obtained parameters in patients with PH remain sparse and exist primarily for pre-capillary PH [4, 13, 14]. Thus, this study aimed to evaluate associations of RA and RV strain values assessed by 2D STE with invasively measured hemodynamic parameters in patients PH from an all-comer cohort of a cardiology department.

## Methods

### Study design and population

This study is a retrospective analysis of a single-center database of the Cardiology Department, Charité-Universitätsmedizin Berlin, Campus Mitte, Germany. 78 patients were enrolled in the study in the years 2013 to 2015. All underwent hemodynamic assessment by left and right heart catheterization and had a comprehensive TTE examination within 24 h periprocedural (median 3.2 h). A comprehensive medical history was collected in all study participants by clinical interview at admission and by medical records. Exclusion criteria were ongoing mechanical ventilation, presence of severe mitral valve stenosis, mechanical prosthetic heart valves, heart transplantation, and insufficient image quality. PH was defined as an invasively measured mPAP of ≥25 mmHg at rest [1]. We distinguished between pre-capillary PH, which is defined as an elevated mPAP of ≥25 mmHg but normal PCWP ≤15 mmHg, and post-capillary PH, where mPAP and PCWP are both elevated (mPAP ≥25 mmHg and PCWP > 15 mmHg) [1]. Combined pre- and post-capillary PH was defined as an elevated PCWP and diastolic pressure gradient (defined as the difference between diastolic PAP and PCWP) of ≥7 mmHg [1].

The study has been approved by the institutional ethics committee of the Charité-Universitätsmedizin Berlin (EA4/067/13) and complied with the Declaration of Helsinki.

### Echocardiographic examination

A comprehensive TTE was performed using a Vivid 7 Dimension or Vivid E9 (GE Vingmed, Horton, Norway; M4S or M5S 1.5–4.5 MHz transducer) ultrasound systems. All investigators in the echocardiography laboratory were blinded for invasively obtained data. Standard left and right heart echocardiographic parameters were obtained according to the recommendations of the American Society of Echocardiography (ASE) and European Association of Cardiovascular Imaging (EACVI) [15, 16]. These included measurement of tricuspid annular plane systolic excursion (TAPSE) with M-Mode and RV peak systolic velocity (RV-S′) with Doppler tissue imaging (DTI). Three beats of each view were recorded for offline analysis (EchoPac PC, GE Vingmed). RA area and RV diameter (basal, mid and apico-basal) were measured directly from 2D images according to the recommendations of ASE and EACVI [15, 16]. Endocardial borders of the RV were traced in an apical four-chamber view for detection of end-diastolic and end-systolic RV area (RVED and RVES area, respectively) and RV fractional area change (FAC) was calculated. Left ventricular ejection fraction (LVEF) was calculated using biplane Simpson’s method and Auto-EF tool (GE Vingmed) as previously described [17].

### 2D speckle tracking strain analysis

For longitudinal strain analyses, multiple standard 2D ultrasound images were recorded with a frame rate between 60 and 80 frames per second and digitally stored for offline analysis (EchoPac PC, GE Vingmed). After manual tracing of the endocardial border of the RA, RV and left ventricle (LV) [18, 19], the region of interest (ROI) was semi-automatically determined. The ROI was manually adjusted for optimal tracking quality when necessary. For all strain analyses, an arithmetic mean value of three individual measurements was used.

**Fig. 1:**
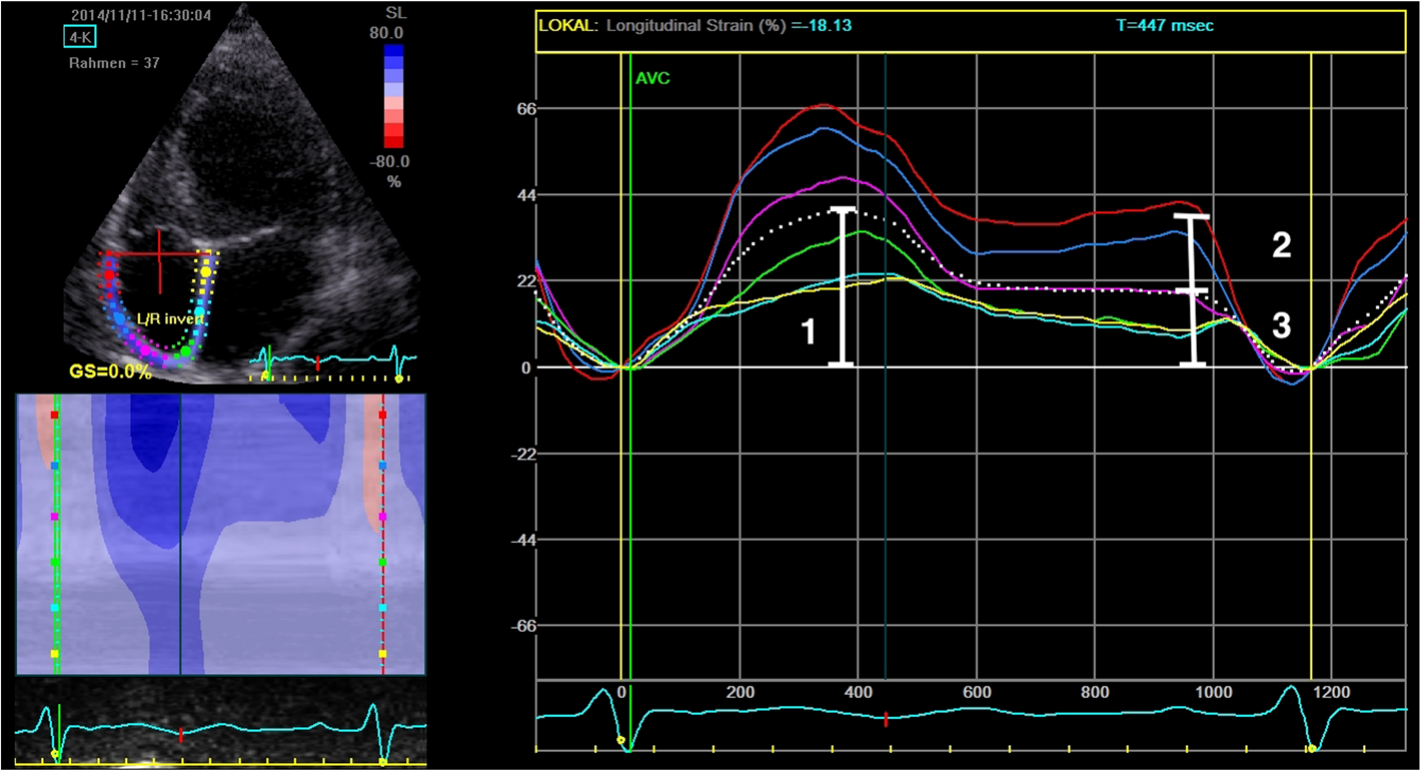
Representative example of speckle tracking echocardiography (2D STE-) derived longitudinal strain of the right atrium obtained in the apical 4-chamber view. Dotted white curve indicates mean of all segments. The maximum excursion of the average strain curve [1] symbolizes the maximum longitudinal strain and RA reservoir function (RAS). RA conduit function was calculated from the difference between RAS and the end of the conduit phase in early diastole [2] and RA contractile function was derived from the difference between the endpoint of conduit phase and the peak RA contraction [3]

For phasic RA strain assessment, a RV-focused four chamber view, avoiding foreshortening of the RA, was chosen. Phasic RA strain analyses were performed using 2D speckle tracking echocardiography [19] identifying strain values during the three phases of the RA cycle from the resulting strain curves (see Fig. 1 for illustration). The maximum excursion of the average strain curve (dotted line in Fig. 1) symbolizes the maximum longitudinal strain and RA reservoir function (RAS). Next, RA conduit function was calculated from the difference of strain values of conduit phase in early diastole (onset of the atrial contraction) and RAS, while RA contraction function was derived from the difference between the endpoint of conduit phase (at the onset of atrial contraction) and the peak negative RA strain shortening (RA contraction, see Fig. 1) [19]. In patients with atrial fibrillation, RA conduit and contractile function was not obtained.

**Fig. 2:**
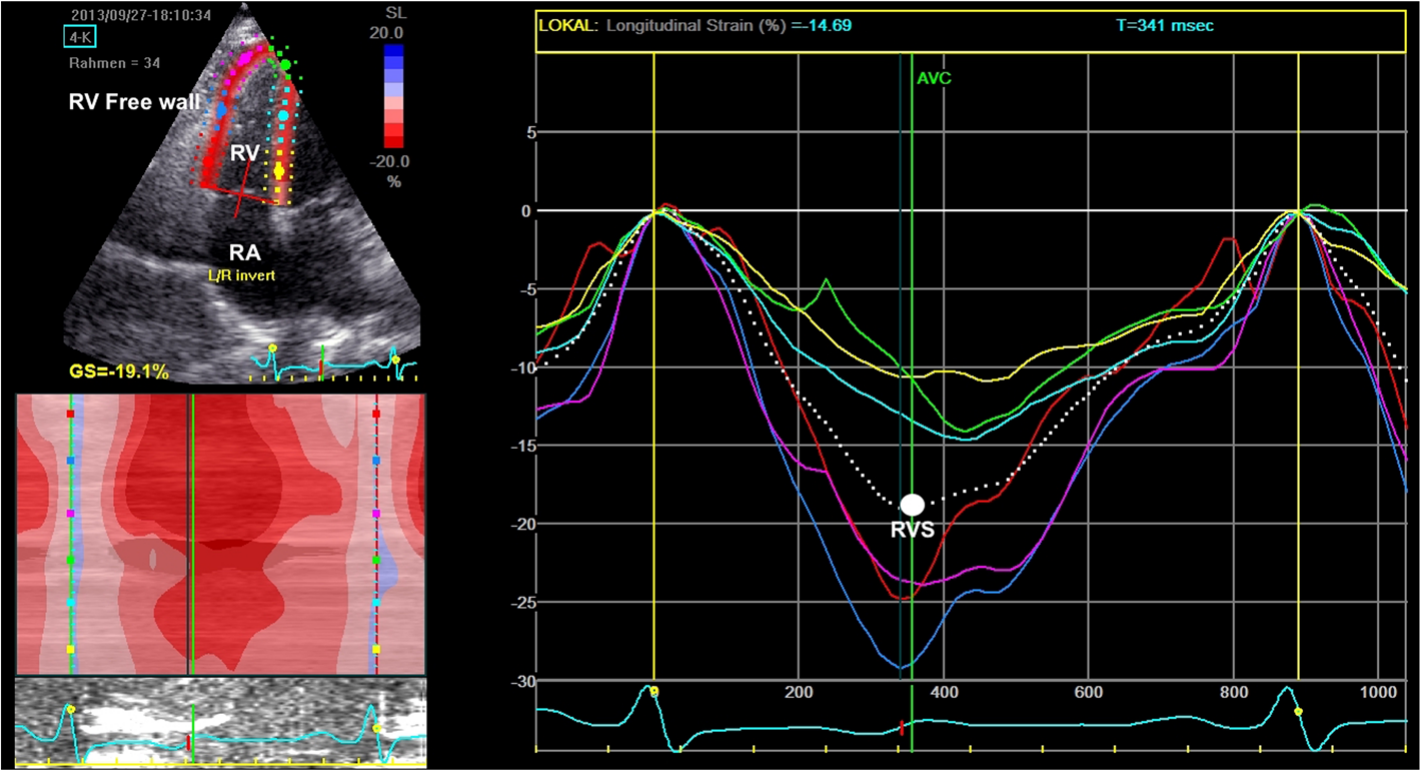
Representative example of speckle tracking echocardiography (2D STE-) derived longitudinal strain of the right ventricle obtained in the apical 4-chamber view. Dotted white curve indicates mean of all segments, where RVS is derived from maximum excursion. The RV Free wall consists of three segments, which are colored red, dark blue and pink in the STE analysis

For longitudinal RV strain analyses, a RV-focused apical four-chamber view was used. We analyzed RV strain in four segments, three of them being part of the RV free wall (basal, mid and apical segment) as well as the inter-ventricular septum (see Fig. 2 for illustration). Combining the average peak systolic strain of all RV segments resulted in an average RV strain (RVS), while RV free wall comprises the average strain of all three free wall segments.

LV global longitudinal peak systolic strain (LV-GLPS) was measured semi-automatically by strain analyses of the apical four-, three- and two-chamber-view as previously described [17].

### Cardiac catheterization

All patients had a clinical indication for a combined right and left heart catheterization. Cardiac catheterization measurements were performed as previously described [17]. Briefly, arterial and venous accesses were established by inserting sheaths in the femoral artery and vein, respectively. Then a fluid-filled pigtail catheter (6 French, Cordis, a Cardinal Health company) was placed in the LV for LV end-diastolic pressure (LVEDP) measurements. For measurements of right heart pressures, pulmonary artery pressure (PAP) and PCWP a Corodyn P1 right heart catheter (B. Braun, Germany) was used. Exact wedge position was verified by changes in pressure waveforms and fluoroscopy. Cardiac index, systemic vascular resistance (SVR) and pulmonary vascular resistance (PVR) were calculated as previously described [17]. All patients were hemodynamically stable at the time of the examination. All measurements were acquired during an unforced end expiratory breath-hold and were completed before injection of contrast agents into the left ventricle or coronary arteries was performed.

### Intra- und inter-observer agreement

To determine intra- and interobserver variability for RA and RV strain parameters, a second experienced cardiologist, who was blinded to previously obtained data, separately measured RA and RV strain parameters of 20 randomly selected patients. Intraclass correlation coefficients (ICC) were calculated for RVS, RAS, RA conduit and contractile function.

### Statistical analysis

Statistical analysis was performed using SPSS (Version 25, IBM Corporation). For continuous variables, results are expressed as arithmetic mean and standard deviation (SD) or, if not normally distributed variables, as median with interquartile ranges (IQR = 25th–75th percentile). Accordingly, Student’s t-test or Mann-Whitney U test was used for comparison of groups. Categorical data are presented as absolute numbers and respective percentages. Categorical variables were compared by chi-square tests. For correlation analyses between variables, Pearson’s or Spearman’s correlation coefficient were calculated as applicable. A *p*-value < 0.05 was considered statistically significant.

## Results

### Study population

A total of 78 patients were enrolled in the present study. 45 patients presented with PH, while 33 patients had no signs of PH (non-PH). Within the PH group, 39 had post-capillary, five had combined pre- and post-capillary PH, and one had pre-capillary PH. Baseline clinical and demographic characteristics of all patients and individual groups of PH and non-PH patients are listed in Table 1. None of the patients had tricuspid or pulmonary valve stenosis.

**Table 1:** Baseline characteristics

Characteristic	All (***n*** = 78)	PH (***n*** = 45)	Non-PH (***n*** = 33)	***p***-value
Number of patients, n (%)	78	45 (57.7)	33 (42.3)	
Age, years	72.6 ± 12.8	75.1 ± 11.5	69.3 ± 13.9	**0.049**
Gender female/male, n (%)	17 (22.0) / 61 (78.0)	16 (35.6) / 29 (64.4)	32 (97.0)/ 1 (3.0)	**0.001**
BMI, kg/m^2^	26.0 (24.0–29.4)	26.7 (24.0–29.5)	24.9 (24.0–27.8)	0.107
Heart rate, /min	71.0 ± 12.3	72.6 ± 13.4	68.8 ± 10.2	0.181
SBP, mmHg	137.0 (118.7–150.0)	137.0 (116.0–149.0)	137.0 (121.5–153.5)	0.671
DBP, mmHg	68.4 ± 12.3	68.9 ± 13.3	67.8 ± 11.1	0.693
AF, n (%)	21 (26.9)	16 (35.6)	5 (15.2)	**0.045**
Diabetes, n (%)	23 (29.5)	17 (37.8)	6 (18.2)	0.061
Arterial hypertension, n (%)	56 (71.8)	35 (77.8)	21 (63.6)	0.170
CAD, n (%)	33 (42.3)	24 (53.3)	9 (27.3)	**0.021**
Previous MI, n (%)	13 (16.7)	10 (22.2)	3 (9.1)	0.124
Previous CABG, n (%)	7 (9.0)	6 (13.3)	1 (3.0)	0.116
Previous TIA/stroke, n (%)	5 (6.4)	3 (6.7)	2 (6.1)	0.914
PAD, n (%)	8 (10.3)	6 (13.3)	2 (6.1)	0.296
Previous bAVR, n (%)	4 (5.1)	3 (6.7)	1 (3.0)	0.472
PM, n (%)	14 (17.9)	10 (22.2)	4 (12.1)	0.251
ICD, n (%)	2 (2.6)	2 (4.4)	0	0.220
COPD, n (%)	16 (20.5)	13 (28.9)	3 (9.1)	**0.032**

Data are expressed as mean ± SD, median with IQR or absolute numbers and respective percentages. AF, atrial fibrillation; BMI, body mass index; SBP, systolic blood pressure; DBP, diastolic blood pressure; CAD, coronary artery disease; MI, myocardial infarction; CABG, coronary artery bypass grafting; TIA, transient ischemic attack; PAD, peripheral artery disease; bAVR, bioprosthetic aortic valve replacement; PM, pace maker; ICD, implantable cardioverter defibrillator; COPD, chronic obstructive pulmonary disease.

### Echocardiographic and invasive characteristics

Detailed cardiac catheterization and echocardiographic data of all patients and individual groups of PH and non-PH patients are presented in Table 2. Invasively assessed mPAP, PWCP, and LVEDP were significantly higher in PH compared to non-PH patients while cardiac index was significantly lower in the PH group (Table 2). Patients with PH had significantly larger RA and RV end-systolic area (RVES area) compared to non-PH patients. TAPSE, RV-S′, LVEF, and LV-GPLS were significantly lower in the PH group (Table 2).

**Table 2:** Echocardiographic and cardiac catheterization data

Parameter	All (***n*** = 78)	PH (***n*** = 45)	Non-PH (***n*** = 33)	***p***-value
**Echocardiographic data**				
RA area, cm^2^	16.9 (13.0–24.9)	20.2 (15.8–27.0)	14.2 (12.3–18.0)	**0.003**
RVED area, cm^2^	21.9 (17.9–25.6)	22.1 (18.7–27.3)	20.4 (15.8–25.4)	0.130
RVES area, cm^2^	12.2 (9.9–15.6)	13.1 (10.8–17.1)	10.7 (9.3–13.4)	**0.022**
FAC, %	41.3 (32.7–49.1)	41.0 (33.0–48.4)	43.5 (32.3–53.3)	0.749
RV base diameter, mm	38.2 (33.5–42.9)	39.6 (34.6–43.9)	36 (30.3–40.7)	0.104
RV mid diameter, mm	26.6 (22.1–32.7)	29.3 (22.4–34.6)	24.8 (21.5–31.5)	0.146
RV apex-base diameter, mm	77.4 (66.3–84.2)	77.6 (66.3–84.9)	75.8 (66.4–81.8)	0.402
RVOT mm	32.3 ± 4.3	32.5 ± 4.1	31.8 ± 4.5	0.54
TAPSE, mm	19.8 ± 5.6	17.8 ± 5.4	22.3 ± 4.7	**< 0.001**
RV-S′, cm/s	10.9 ± 3.1	10.0 ± 3.1	12.2 ± 2.6	**0.003**
RVOT VTI	13.1. ± 4.0	12.0 ± 4.2	14.6 ± 3.2	**0.006**
RV-e‘cm/s	10.7 ± 3.3	10.6 ± 3.8	10.7 ± 2.7	**0.76**
RV-a’cm/s	12.7 ± 5.6	11.3 ± 6.4	14.2 ± 4.3	**0.045**
LV ejection fraction, %	45.0 ± 13.4	39.0 ± 12.4	52.4 ± 11.0	**< 0.001**
*≥ 55, n (%)*	25 (32.1)	8 (17.8)	17 (51.5)	
*45–54, n (%)*	17 (21.8)	8 (17.8)	9 (27.3)	
*30–44, n (%)*	27 (34.6)	21 (46.7)	6 (18.2)	
*≤ 30, n (%)*	9 (11.5)	8 (17.8)	1 (3.0)	
LV-GPLS	−13.6 ± 5.5	−11.2 ± 5.3	−16.5 ± 4.3	**< 0.001**
Aortic valve regurgitation, n (%)				0.122
*none*	42 (53.8)	20 (44.4)	22 (66.7)	
*mild*	25 (32.1)	16 (35.6)	9 (27.3)	
*moderate*	9 (11.5)	8 (17.8)	1 (3)	
severe	2 (2.6)	1 (2.2)	1 (3)	
Aortic valve stenosis, n (%)				0.544
*none*	54 (69.2)	29 (64.4)	25 (75.8)	
*mild*	1 (1.3)	1 (2.2)	0	
*moderate*	1 (1.3)	1 (2.2)	0	
*severe*	22 (28.2)	14 (31.1)	8 (24.2)	
Mitral valve regurgitation, n (%)				**0.002**
*none*	16 (20.5)	8 (17.8)	8 (24.2)	
*mild*	27 (34.6)	9 (20.0)	18 (54.5)	
*moderate*	30 (38.5)	25 (55.6)	5 (15.2)	
*severe*	5 (6.4)	3 (6.7)	2 (6.1)	
Mitral valve stenosis, n (%)				0.675
*none*	75 (96.2)	43 (95.6)	32 (97.0)	
*mild*	2 (2.6)	1 (2.2)	1 (3.0)	
*moderate*	1 (1.3)	1 (2.2)	0	
Tricuspid valve regurgitation, n (%)				**< 0.001**
*none*	18 (23.1)	5 (11.1)	13 (39.4)	
*mild*	31 (39.7)	16 (35.6)	15 (45.5)	
*moderate*	21 (26.9)	16 (35.6)	5 (15.2)	
*severe*	8 (10.3)	8 (17.8)	0	
Pulmonary valve regurgitation, n (%)				0.060
*none*	50 (64.1)	24 (53.3)	26 (78.8)	
*mild*	27 (34.6)	20 (44.4)	7 (21.2)	
*severe*	1 (1.3)	1 (2.2)	0	
**Catheterization data**				
mPAP, mmHg	28.0 (21.0–36.0)	35.0 (30.0–42.5)	20.0 (17.0–22.5)	**< 0.001**
PCWP, mmHg	20.0 ± 9.0	25.9 ± 6.8	11.8 ± 3.9	**< 0.001**
Cardiac index, ml/min/m2	2.2 ± 0.6	2.0 ± 0.6	2.4 ± 0.4	**0.001**
LVEDP, mmHg	20.0 (14.5–27.5)	24.5 (21.0–30.7)	15.0 (13.0–17.5)	**< 0.001**
SVR, dyn x sec x cm^−5^	1619.5 (1345–2029.0)	1712.0 (1344–2178.0)	1536.0 (1334–1794.0)	0.189
PVR, dyn x sec x cm^−5^	205.5 (138.2–251.0)	212.0 (164.5–288.5)	180.0 (111.0–238.5)	0.056

Data are expressed as mean ± SD, median with IQR, or absolute numbers and respective percentages. PH, pulmonary hypertension; RA, right atrial; RV, right ventricular; RVED and RVES, RV end diastolic and end systolic; FAC, fractional area change; TAPSE, tricuspid annular plane systolic excursion; RV-S′, RV peak systolic velocity; LV-GLPS, left ventricular global longitudinal peak systolic strain; LVEDP, left ventricular enddiastolic pressure; mPAP, mean pulmonary artery pressure; PCWP, pulmonary capillary wedge pressure; SVR, systemic vascular resistance; PVR, pulmonary vascular resistance

Due to the presence of atrial fibrillation at examination, assessment of RA conduit and contractile function was limited to 59 patients (PH *n* = 30, non-PH *n* = 29). RA and RV strain was reduced in the PH group but the difference did not reach statistical significance except for average mid RV strain (Table 3).

**Table 3:** Right atrial (RA) and right ventricular (RV) strain parameters

Parameter	All (n = 78)	PH (n = 45)	Non-PH (n = 33)	***p***-value
**RA strain parameters**				
RAS, %	19.5 (10.4–26.9)	14.8 (7.5–26.3)	21.3 (15.3–29.9)	0.060
Conduit function, %*	10.0 (5.8–14.7)	7.5 (3.7–14.7)	11.7 (7.6–15.6)	0.063
Contractile function, %*	11.8 (8.4–15.8)	11.1 (5.3–13.9)	13.5 (9.2–16.5)	0.870
**RV strain parameters**				
RVS, %	−12.3 ± 6.5	−11.5 ± 6.3	−13.6 ± 6.8	0.196
Basal strain, %	−21.4 ± 9.3	−20.4 ± 10.7	− 23.0 ± 6.1	0.210
Mid strain, %	−19 ± 7.3	−17.4 ± 7.8	− 21.6 ± 5.5	**0.019**
Apical strain, %	− 9.3 (− 16.9 - -5.9)	−8.8 (− 15.2 - -5.8)	−11.1 (− 18.7 - -6.6)	0.350
Free Wall, %	−17.2 ± 6.5	−16.15 ± 7.1	−19.0 ± 4.8	0.072

Data are expressed as mean ± SD or median with IQR. PH, pulmonary hypertension; RAS, mean right atrial reservoir strain; RVS, mean right ventricular strain; * = atrial fibrillation, RA conduit and contraction function was only calculated for 59 patients (PH *n* = 30, non-PH *n* = 29).

### Associations between echocardiographic standard and deformation parameters and invasive characteristics

Associations between echocardiographic and invasively assessed parameters are listed in Table 4. There were no significant associations between echocardiographically assessed right heart parameters and invasively measured mPAP and PCWP in non-PH patients. In PH patients, RAS showed a significant inverse association with mPAP (r = − 0.470, *p* = 0.001) and with PCWP (r = − 0.296, *p* = 0.048), while RA conduit and contractile function did not show any significant associations with mPAP or PCWP.

**Table 4:** Correlation analysis of mean right atrial (RA) and right ventricular (RV) strain parameters with invasively obtained hemodynamic parameters

	PH (n = 45)				Non-PH (n = 33)			
**Parameter**	**mPAP**		**PCWP**		**mPAP**		**PCWP**	
	correlation coefficient	*p* value	correlation coefficient	p value	correlation coefficient	p value	correlation coefficient	p value
RA area	0.297	0.050	0.189	0.219	0.043	0.814	0.281	0.126
RVED area	**0.382**	**0.012**	**0.389**	**0.010**	0.106	0.598	0.142	0.489
RVES area	0.271	0.078	**0.338**	**0.027**	− 0.026	0.899	0.026	0.900
FAC	0.089	0.572	0.130	0.407	0.157	0.434	0.031	0.882
RV base diameter	**0.346**	**0.023**	**0.318**	**0.038**	0.051	0.792	0.213	0.277
RV mid diameter	0.251	0.104	0.188	0.227	−0.182	0.343	0.182	0.355
RV apex-base diameter	0.077	0.626	0.104	0.507	− 0.073	0.705	− 0.258	0.184
TAPSE	**0.378**	**0.010**	0.181	0.233	0.245	0.154	0.310	0.085
RV-S′	**0.323**	**0.037**	0.166	0.294	0.453	0.10	−0.069	0.717
RVOT VTI	**−0.364**	**0.019**	−0.222	0.16	0.377	0.044	0.059	0.77
RV-e‘	0.200	0.20	0.132	0.40	**0.455**	**0.009**	0.269	0.14
RV-a‘	**−0.455**	**0.009**	−0,275	0.13	−0,104	0.58	−0.111	0.56
**RA strain parameters**								
RAS	**−0.470**	**0.001**	**−0.296**	**0.048**	−0.017	0.923	−0.177	0.334
Conduit function *	−0.342	0.065	−0.171	0.366	0.259	0.175	−0.019	0.924
Contractile function *	−0.335	0.071	−0.242	0.198	0.045	0.818	−0.018	0.927
**RV strain parameters**								
RVS	**0.490**	**0.001**	**0.365**	**0.015**	−0.130	0.520	−0.052	0.802
Basal strain	**0.395**	**0.009**	0.131	0.404	0.062	0.763	−0.307	0.135
mid strain	**0.443**	**0.003**	0.260	0.092	−0.180	0.378	−0.024	0.910
Apical strain	**0.461**	**0.002**	**0.348**	**0.220**	−0.298	0.139	0.242	0.244
Free Wall	**0.466**	**0.002**	0.258	0.094	−0.277	0.265	−0.061	0.773

PH, pulmonary hypertension; mPAP, mean pulmonary artery pressure; PCWP, pulmonary capillary wedge pressure; RA, right atrial; RV, right ventricular; RVED and RVES, RV end diastolic and end systolic; FAC, fractional area change; TAPSE, tricuspid annular plane systolic excursion; RV-S′, RV peak systolic velocity; RAS, mean right atrial reservoir strain; * = atrial fibrillation, RA conduit and contraction function was only calculated for 59 patients (PH n = 30, non-PH n = 29); RVS = mean right ventricular strain

Concerning RV parameters, moderate associations between mPAP and RVS (r = 0.490, p = 0.001), RV free wall strain (r = 0.466, *p* = 0.002), RV mid strain(r = 0.444, *p* = 0.003) or RV apical strain (r = 0.461, p = 0.002) were found in PH patients. Associations between mPAP and TAPSE (r = 0.378, *p* = 0.010), RV-S′ (r = 0.323, *p* = 0.037), RVED area (r = 0.382, *p* = 0.012), or RV base diameter (r = 0.346, *p* = 0.023) were weak to moderate.

There were significant associations between PCWP and RVED area (r = 0.389, *p* = 0.019), RVES area (r = 0.338, *p* = 0.027), RVS (r = 0.365, *p* = 0.015), RV base diameter (r = 0.318, *p* = 0.038), or RV apical strain (r = 0.348, *p* = 0.220). While there was a significant difference in RA area between patients with PH and without PH, there was no significant association of RA area with mPAP or PCWP.

RVOT VTI and RV-a’ showed weak to moderate associations with RAS and RVS (RVOT VTI: r = 0.244, *p* = 0.042; r = − 0.306, p = 0.015 for RAS and RVS; RV-a’: r = 0.405, *p* = 0.001; and r = − 0,256, p = 0,055 for RAS and RVS; respectively). Some of the clinical characteristics with possible impact on PH diagnosis were different between groups; however, none of these parameters showed significant associations with the diagnosis of PH in a multivariate logistic regression model (see Supplement, Tables S1 and S2).

### Intra- and inter-observer agreement

The ICC for intra-observer agreement was 0.86 (95% confidence interval [CI] 0.68–0.94) for RAS, 0.88 (CI 0.73–0.95) for RA conduit function, 0.86 (CI 0.68–0.94) for RA contractile function and 0.75 (CI 0.48–0.89) for RVS. ICC for inter-observer agreement was 0.92 (CI 0.81–0.97), 0.89 (CI 0.75–0.95), 0.82 (CI 0.61–0.92) and 0.91 (CI 0.8–0.96), respectively.

## Discussion

In the present study, we analyzed echocardiographically and invasively assessed hemodynamic parameters in a cohort of all-comer patients with and without PH. We demonstrated significant associations between right heart deformation parameters (2D STE-derived RA and RV strain) and invasively assessed mPAP and PCWP in patients with predominantly post-capillary PH, while no significant associations were found in non-PH patients. To our knowledge, this is the first study providing a comprehensive analysis of right heart strain parameters in comparison with invasive hemodynamic measurements in patients with predominantly post-capillary PH.

Imaging of the right heart, especially of the RA, by TTE often remains challenging. Since we mainly examined patients with post-capillary PH which also have several left heart and valve diseases, imaging of the right heart can become even more challenging. Still 2D TTE remains the most widely accessible and used diagnostic tool in cardiology and additional methods like STE are needed to extend the information derived from TTE in different clinical settings. Several studies such as Padeletti et al. [9] and Peluso et al. [10] have shown that STE is a valuable and practicable method for RA and RV function assessment.

PH causes an increase of RV afterload with consecutive elevation of RV pressures, which require adaptation methods of the RV such as intensifying contraction and increasing muscle mass (myocardial hypertrophy). This can result in impairment of RV systolic function, making parameters for RV systolic function such as RVS some of the most valuable predictors for outcome in patients with PH [20]. Previous studies [13, 14, 21] reported impaired RA and RV strain values in patients with pre-capillary PH. Similarly, in our study RA strain values were lower and RV strain values higher in PH patient compared to non-PH patient. While PH patients in our study consisted of an all-comer cohort and mainly represented patients with post-capillary PH caused by left heart diseases, previous studies that reported significant impairment of RA and RV strain mainly focused on patients with idiopathic or scleroderma-associated PH [13] and/or chronic thromboembolic pulmonary hypertension [14, 21]. Precapillary PH may cause an earlier onset and more severe impairment of right heart function. This makes comparisons of these studies with our findings difficult as individual entities of PH and different comorbidities may have a specific impact on RA and RV function.

Standard echocardiographic right heart function parameters used in clinical routine comprise TAPSE, RV-S′, or FAC. In our study TAPSE and RV-S′ showed a significant difference between PH and non-PH patients with lower levels for both parameters in the presence of PH while RA and RV strain parameters did not differ between PH and non-PH patients. Still there was a trend towards lower RAS and RV free wall levels in PH patients which should be validated in a larger sample size. Concerning associations with hemodynamic parameters (mPAP and PCWP), RAS and RVS showed stronger associations in patients with PH compared to TAPSE and RV-S′. Other studies recently described that 2D STE of the RV free wall has been shown to be a feasible and more accurate parameter for predicting outcome and cardiovascular events in patients with predominantly pre-capillary PH [11, 21, 22]. This superiority is mainly explained by the global assessment of right heart function by 2D STE and higher sensitivity of 2D STE while e.g. TAPSE remains a localized, one-dimensional measurement of the tricuspid annular motion [21, 22]. This suggests that right heart 2D STE may be a more accurate method to detect functional myocardial impairment compared to standard echocardiographic parameters.

Long-term pressure overload and adaptation due to LV dysfunction and PH may result in RV diastolic dysfunction and affect the pressure gradient between RA and RV [5, 23], which leads to RA remodeling, enlargement and dysfunction [24]. Assessment of RA function can be implemented in TTE by using 2D STE. In our study, RAS showed a moderate inverse association with mPAP and a weak inverse correlation with PCWP in patients with PH. Regarding RA conduit and contractile function, associations with mPAP and PCWP did not reach statistical significance, which may be a result of the small sample size due to the exclusion of AF patients for these two parameters. The reported moderate associations of RAS and RVS with invasive pulmonary pressures may be explainable by the fact that RAS and RVS are, next to an increase of RV afterload, significantly associated with geometric and structural properties of the RA and the RV (such as hypertrophy and remodeling processes) with impact on compliance and wall tension, as well as on systolic RV and LV performance [8, 25]. The results of our study, however, demonstrate that RAS may be a valuable noninvasive parameter for monitoring right heart function of PH patients. Sato et al. [3] and Alenezi et al. [5] have shown that RA reservoir, conduit and contractile function are independent predictors for mortality and hospitalization in patients with PH (WHO group 1 and 4). Further studies are wanted to validate whether the predictive value of RA function parameters is also applicable in patients with post-capillary PH.

### Limitations

The present study has several limitations. First, it is a single-center analysis with a relatively small patient cohort; while there were significant associations with invasively and non-invasively assessed parameters, correlation strength and significance may be underestimated.

Second, our cohort consisted of all-comers of the cardiology department and many patients had multiple other cardiovascular diseases besides PH, which may have a relevant impact on the results. On the contrary, this makes our findings more applicable and suitable for daily clinical routine. Since the etiology of PH in our patient cohort was predominantly post-capillary, these results might not be applicable for pre-capillary PH. As mentioned above, most of previous studies in this field focussed on precapillary PH excluding patients with left heart diseases. While long-term LV dysfunction may finally lead to post-capillary PH, it may take longer to cause measurable functional impairments of the RA and RV and some of our patients might not even have reached that point yet [26, 27].

Third, this study did not include follow-up measurements. Future studies are needed to investigate the prognostic value of 2D STE derived RA and RV parameters for mortality and long-term outcome in patients with post-capillary PH.

## Conclusion

Our study showed significant associations between invasively assessed hemodynamic parameters and right heart strain parameters, particularly RAS and RVS, in patients with predominantly post-capillary PH. The analysis of RA and RV strain may be useful for monitoring of right heart function in these patients. Prospective studies evaluating RA and RV strain alterations in patients with different entities of PH are needed to clarify the prognostic value of these parameters.

## Supplementary information

**Additional file 1: Table S1.** Associations of diverse clinical characteristics and RV strain with PH; multivariate regression analysis. **Table S2.** Associations of diverse clinical characteristics and RA strain with PH; multivariate regression analysis.

## Data Availability

The data that support the findings of this study are available from the corresponding author upon reasonable request.

## Abbreviations

RA: Right atrium / atrial
RV: Right ventricle / ventricular
2D STE: 2D speckle tracking echocardiography
PH: Pulmonary hypertension
mPAP: Mean pulmonary arterial pressure
PCWP: Pulmonary capillary wedge pressure
RAS: Average peak systolic right atrial strain / right atrial reservoir function
RVS: Average peak systolic right ventricular strain
PAP: Pulmonary artery pressure
TTE: Transthoracic echocardiography
TAPSE: Tricuspid annular plane systolic excursion
RV-S′: Right ventricle peak systolic velocity
DTI: Doppler tissue imaging
ASE: Amercian Society of Echocardiography
EACVI: European Association of Cardiovascular Imaging
RVED area: Right ventricular end-diastolic area
RVES area: Right ventricular end-systolic area
FAC: Right ventricle fractional area change
LVEF: Left ventricular ejection fraction
LV: Left ventricle
ROI: Region of interest
LV-GLPS: Left ventricular global longitudinal peak systolic strain
LVEDP: Left ventricular end-diastolic pressure
SVR: Systemic vascular resistance
PVR: Pulmonary vascular resistance
ICC: Intraclass correlation coefficient
SD: Standard deviation
IQR: Interquartile ranges
Non-PH: Patients without pulmonary hypertension
RVES area: Right ventricular end-systolic area
CI: Confidence interval
CTEPH: Chronic thrombembolic pulmonary hypertension

## References

[1] Galie N, Humbert M, Vachiery JL, Gibbs S, Lang I, Torbicki A, et al. 2015 ESC/ERS guidelines for the diagnosis and treatment of pulmonary hypertension. Rev Esp Cardiol (Engl Ed). 2016;69(2):177. 10.1016/j.rec.2016.01.002 26837729

[2] Haeck ML, Scherptong RW, Marsan NA, Holman ER, Schalij MJ, Bax JJ (2012). Prognostic value of right ventricular longitudinal peak systolic strain in patients with pulmonary hypertension. Circ Cardiovasc Imaging.

[3] Sato T, Tsujino I, Ohira H, Oyama-Manabe N, Ito YM, Yamada A (2015). Right atrial volume and reservoir function are novel independent predictors of clinical worsening in patients with pulmonary hypertension. J Heart Lung Transplant.

[4] Querejeta Roca G, Campbell P, Claggett B, Solomon SD, Shah AM. Right Atrial Function in Pulmonary Arterial Hypertension. Circ Cardiovasc Imaging. 2015;8(11):e003521; discussion e. 10.1161/CIRCIMAGING.115.003521 PMC4629509 26514759

[5] Alenezi F, Mandawat A, Il'Giovine ZJ, Shaw LK, Siddiqui I, Tapson VF (2018). Clinical utility and prognostic value of right atrial function in pulmonary hypertension. Circ Cardiovasc Imaging.

[6] Brand A, Bathe M, Oertelt-Prigione S, Seeland U, Rucke M, Regitz-Zagrosek V (2018). Right heart function in impaired left ventricular diastolic function: 2D speckle tracking echocardiography-based and Doppler tissue imaging-based analysis of right atrial and ventricular function. Echocardiography.

[7] Padeletti M, Cameli M, Lisi M, Zaca V, Tsioulpas C, Bernazzali S (2011). Right atrial speckle tracking analysis as a novel noninvasive method for pulmonary hemodynamics assessment in patients with chronic systolic heart failure. Echocardiography.

[8] Brand A, Bathe M, Hubscher A, Baldenhofer G, Hattasch R, Seeland U, et al. Normative reference data, determinants, and clinical implications of right atrial reservoir function in women assessed by 2D speckle-tracking echocardiography. Echocardiography. 2018. 10.1111/echo.14092 29962056

[9] Padeletti M, Cameli M, Lisi M, Malandrino A, Zaca V, Mondillo S (2012). Reference values of right atrial longitudinal strain imaging by two-dimensional speckle tracking. Echocardiography.

[10] Peluso D, Badano LP, Muraru D, Dal Bianco L, Cucchini U, Kocabay G (2013). Right atrial size and function assessed with three-dimensional and speckle-tracking echocardiography in 200 healthy volunteers. Eur Heart J Cardiovasc Imaging.

[11] Motoji Y, Tanaka H, Fukuda Y, Ryo K, Emoto N, Kawai H (2013). Efficacy of right ventricular free-wall longitudinal speckle-tracking strain for predicting long-term outcome in patients with pulmonary hypertension. Circ J.

[12] Fukuda Y, Tanaka H, Sugiyama D, Ryo K, Onishi T, Fukuya H (2011). Utility of right ventricular free wall speckle-tracking strain for evaluation of right ventricular performance in patients with pulmonary hypertension. J American Soc Echocardiography.

[13] Bhave NM, Visovatti SH, Kulick B, Kolias TJ, McLaughlin VV (2017). Right atrial strain is predictive of clinical outcomes and invasive hemodynamic data in group 1 pulmonary arterial hypertension. Int J Cardiovasc Imaging.

[14] Fukuda Y, Tanaka H, Ryo-Koriyama K, Motoji Y, Sano H, Shimoura H (2016). Comprehensive functional assessment of right-sided heart using speckle tracking strain for patients with pulmonary hypertension. Echocardiography.

[15] Rudski LG, Lai WW, Afilalo J, Hua L, Handschumacher MD, Chandrasekaran K, et al. Guidelines for the echocardiographic assessment of the right heart in adults: a report from the American Society of Echocardiography endorsed by the European Association of Echocardiography, a registered branch of the European Society of Cardiology, and the Canadian Society of Echocardiography. Journal of the American Society of Echocardiography : official publication of the American Society of Echocardiography. 2010;23(7):685–713; quiz 86–8. 10.1016/j.echo.2010.05.010 20620859

[16] Lang RM, Badano LP, Mor-Avi V, Afilalo J, Armstrong A, Ernande L (2015). Recommendations for cardiac chamber quantification by echocardiography in adults: an update from the American Society of Echocardiography and the European Association of Cardiovascular Imaging. Euro Heart J Cardiovasc Imaging.

[17] Hewing B, Theres L, Spethmann S, Stangl K, Dreger H, Knebel F. Left atrial strain predicts hemodynamic parameters in cardiovascular patients. Echocardiography.n/a-n/a. 10.1111/echo.13595 28664601

[18] Voigt JU, Pedrizzetti G, Lysyansky P, Marwick TH, Houle H, Baumann R (2015). Definitions for a common standard for 2D speckle tracking echocardiography: consensus document of the EACVI/ASE/industry task force to standardize deformation imaging. Eur Heart J Cardiovasc Imaging.

[19] Badano LP, Kolias TJ, Muraru D, Abraham TP, Aurigemma G, Edvardsen T (2018). Standardization of left atrial, right ventricular, and right atrial deformation imaging using two-dimensional speckle tracking echocardiography: a consensus document of the EACVI/ASE/industry task force to standardize deformation imaging. Eur Heart J Cardiovasc Imaging.

[20] Ghio S, Klersy C, Magrini G, D'Armini AM, Scelsi L, Raineri C (2010). Prognostic relevance of the echocardiographic assessment of right ventricular function in patients with idiopathic pulmonary arterial hypertension. Int J Cardiol.

[21] Fine NM, Chen L, Bastiansen PM, Frantz RP, Pellikka PA, Oh JK (2013). Outcome prediction by quantitative right ventricular function assessment in 575 subjects evaluated for pulmonary hypertension. Circ Cardiovasc Imaging..

[22] Fukuda Y, Tanaka H, Motoji Y, Ryo K, Sawa T, Imanishi J (2014). Utility of combining assessment of right ventricular function and right atrial remodeling as a prognostic factor for patients with pulmonary hypertension. Int J Cardiovasc Imaging.

[23] Bristow MR, Zisman LS, Lowes BD, Abraham WT, Badesch DB, Groves BM (1998). The pressure-overloaded right ventricle in pulmonary hypertension. Chest.

[24] Willens HJ, Fertel DP, Qin J, Labrador E, Lowery MH (2008). Effects of age and pulmonary arterial hypertension on the different phases of right atrial function. Int J Cardiovasc Imaging.

[25] Lee JH, Park JH (2018). Strain analysis of the right ventricle using two-dimensional echocardiography. J Cardiovasc Imaging.

[26] Vachiery JL, Adir Y, Barbera JA, Champion H, Coghlan JG, Cottin V (2013). Pulmonary hypertension due to left heart diseases. J Am Coll Cardiol.

[27] Fang JC, DeMarco T, Givertz MM, Borlaug BA, Lewis GD, Rame JE (2012). World Health Organization pulmonary hypertension group 2: pulmonary hypertension due to left heart disease in the adult--a summary statement from the pulmonary hypertension Council of the International Society for heart and lung transplantation. J Heart Lung Transplant.

