# Supplementary material for: Associations of 2D speckle tracking echocardiography-based right heart deformation parameters and invasively assessed hemodynamic measurements in patients with pulmonary hypertension
Source: Cardiovasc Ultrasound. 2020 May 14;18:13. doi: 10.1186/s12947-020-00197-z (PMC7227096; doi:10.1186/s12947-020-00197-z)
Supplement: Supplementary file 1 — Additional file 1: Table S1. Associations of diverse clinical characteristics and RV strain with PH; multivariate regression analysis. Table S2. Associations of diverse clinical characteristics and RA strain with PH; multivariate regression analysis. [file 12947_2020_197_MOESM1_ESM.docx]

**SUPPLEMENT**

|  | | |  |  |
| --- | --- | --- | --- | --- |
| **Table S1: Associations of diverse clinical characteristics and RV strain with PH; multivariate regression analysis.** | | | | |
|  | Odds ratio | 95%CI | | *p* |
| aFib | 5.02 | 0.90 to 28.1 | | *.066* |
| AMI | 3.25 | 0.50 to 21.30 | | *0.22* |
| CABG | 0.99 | 0.08 to 12.90 | | *.99* |
| CAD | 1.43 | 0.43 to 4.82 | | *0.56* |
| RVS | 1.04 | 0.95 to 1.15 | | *0.41* |
| aFib, atrial fibrilation; AMI, history of acute myocardial infarction; CABG; history of coronary artery bypass graft; CAD; coronary artery disease; RVS, RV strain. | | | | |

|  | | |  |  |
| --- | --- | --- | --- | --- |
| **Table S2: Associations of diverse clinical characteristics and RA strain with PH; multivariate regression analysis.** | | | | |
|  | Odds ratio | 95%CI | | *p* |
| aFib | 3.11 | 0.87 to 11.2 | | *.082* |
| AMI | 1.75 | 0.34 to 9.07 | | *0.50* |
| CABG | 1.69 | 0.15 to 18.94 | | *0.67* |
| CAD | 2.16 | 0.70 to 6.64 | | *0.178* |
| RAS | 1.01 | 0.97 to 1.04 | | *0.34* |
| aFib, atrial fibrilation; AMI, history of acute myocardial infarction; CABG; history of coronary artery bypass graft; CAD; coronary artery disease; RAS, RA strain. | | | | |
